# Altered Interoceptive Sensibility in Adults With Chronic Tic Disorder

**DOI:** 10.3389/fpsyt.2022.914897

**Published:** 2022-06-21

**Authors:** Ashruta Narapareddy, Michelle R. Eckland, Heather R. Riordan, Carissa J. Cascio, David A. Isaacs

**Affiliations:** ^1^Vanderbilt University, Nashville, TN, United States; ^2^Department of Neurology, Vanderbilt University Medical Center, Nashville, TN, United States; ^3^Department of Pediatrics, Monroe Carell Jr. Children’s Hospital at Vanderbilt, Nashville, TN, United States; ^4^Vanderbilt Kennedy Center, Vanderbilt University, Nashville, TN, United States; ^5^Frist Center for Autism and Innovation, Vanderbilt University, Nashville, TN, United States; ^6^Department of Psychiatry and Behavioral Sciences, Vanderbilt University Medical Center, Nashville, TN, United States

**Keywords:** chronic tic disorder, Tourette syndrome, interoception, interoceptive sensibility, sensory impairment, tics

## Abstract

**Background:**

Interoception refers to the sensing, interpretation, integration, and regulation of signals about the body’s internal physiological state. Interoceptive sensibility is the subjective evaluation of interoceptive experience, as assessed by self-report measures, and is abnormal in numerous neuropsychiatric disorders. Research examining interoceptive sensibility in individuals with chronic tic disorders (CTDs), however, has yielded conflicting results, likely due to methodologic differences between studies and small sample sizes.

**Objective:**

We sought to compare interoceptive sensibility between adults with CTD and healthy controls, adjusting for co-occurring psychiatric symptoms, and to examine the relationship of interoceptive sensibility with other CTD clinical features, in particular, premonitory urge.

**Methods:**

We recruited adults with CTDs and sex- and age-matched healthy controls to complete the Multidimensional Assessment of Interoceptive Awareness, Version 2 (MAIA-2), as well as a battery of measures assessing psychiatric symptoms prevalent in CTD populations. CTD participants additionally completed scales quantifying tic severity, premonitory urge severity, and health-related quality of life. We conducted between-group contrasts (Wilcoxon rank-sum test) for each MAIA-2 subscale, analyzed the effect of psychiatric symptoms on identified between-group differences (multivariable linear regression), and examined within-group relationships between MAIA-2 subscales and other clinical measures (Spearman rank correlations, multivariable linear regression).

**Results:**

Between adults with CTD (*n* = 48) and healthy controls (*n* = 48), MAIA-2 Noticing and Not-Worrying subscale scores significantly differed. After adjusting for covariates, lower MAIA-2 Not-Worrying subscale scores were significantly associated with female sex (β = 0.42, *p* < 0.05) and greater severity of obsessive-compulsive symptoms (β = –0.028, *p* < 0.01), but not with CTD diagnosis. After adjusting for severity of tics and obsessive-compulsive symptoms, a composite of MAIA-2 Noticing, Attention Regulation, Emotional Awareness, Self-Regulation, Body Listening, and Trusting subscales (β = 2.52, *p* < 0.01) was significantly associated with premonitory urge.

**Conclusion:**

Study results revealed three novel findings: adults with CTD experience increased anxiety-associated somatization and increased general body awareness relative to healthy controls; anxiety-associated somatization is more closely associated with sex and obsessive-compulsive symptoms than with CTD diagnosis; and increased general body awareness is associated with greater severity of premonitory urges.

## Introduction

Tics are sudden, recurrent, stereotyped, non-rhythmic movements (motor tics) or vocalizations (vocal tics), often preceded by an unpleasant sensation called a premonitory urge (1). Tourette syndrome (TS) is a neurodevelopmental disorder clinically defined by the presence of multiple motor tics and at least one vocal tic, with emergence of tics before 18 years of age and persistence of tics for at least one year (1). Individuals who experience only motor tics or only vocal tics but fulfill the remainder of the above TS diagnostic criteria are diagnosed with chronic (persistent) motor tic disorder or chronic (persistent) vocal tic disorder, respectively (1). TS, chronic motor tic disorder, and chronic vocal tic disorder exist along a single clinical spectrum (2), with shared underlying genetic architecture (3), and as such, these three disorders are often studied collectively under the label “chronic tic disorders” (CTDs).

While tics, a *discrete* motor phenomenon, and premonitory urges, a *discrete* sensory phenomenon, are the hallmark symptoms of CTDs, individuals with these disorders also manifest *pervasive* motor and sensory abnormalities. Relative to healthy controls, individuals with CTD exhibit altered movement timing (4, 5) and force (6), enhanced reinforcement learning of motor sequences (7), and diminished ability to lateralize fine motor movements (8, 9). Fine motor impairment in children with CTD predicts tic severity in adulthood (10). Sensorimotor integration is aberrant in those with CTD (11–13). The majority of adults and children with CTD endorse heightened sensitivity to commonplace environmental stimuli, a phenomenon termed sensory over-responsivity (14, 15). These clinical and behavioral findings of motor and sensory dysfunction align with neurophysiological (16–18), functional imaging (19–21), and structural imaging (22–25) investigations demonstrating abnormalities at multiple nodes and links of a distributed sensorimotor network in CTDs. Such abnormalities have been identified in primary motor cortex (22, 25, 26), supplementary motor area (19, 25, 26), primary sensory cortex (20, 22), superior parietal cortex (20, 22), insula (21, 27), several basal ganglia structures (20, 27, 28), and white matter tracts within the sensorimotor subcortical region (23, 29). Thus, motor and sensory dysfunction in CTDs is diffuse.

Both motor and sensory function are dynamically intertwined with interoception (30, 31). Interoception refers to the sensing, interpretation, integration, and regulation of signals about the body’s internal physiological state (32–34). Interoception is a continuous, iterative process in which bottom-up afferent signals from the body are integrated in the insula with top-down signals from sensorimotor and frontal cortical regions (33–35). The primary function of interoception is to inform homeostatic drives (33). Numerous higher-order cognitive processes, including memory formation (31), emotion processing (31, 36), and self-representation (30, 31) rely on interoceptive input. Under the widely adopted conceptual framework posited by Garfinkel and Critchley (37), interoception is parsed into three sub-constructs: interoceptive accuracy (objective ability to detect bodily sensations, as assessed by physiological tasks, e.g., heartbeat detection tasks), interoceptive sensibility (subjective evaluation of interoceptive experience, as assessed by self-report measures), and interoceptive awareness (insight into one’s interoceptive accuracy) (32). These inter-related constructs are dissociable (38–40) but appear to share a common neural substrate, the insula (34, 41–43). Individual differences in interoceptive accuracy (34, 41, 42) and interoceptive sensibility (41) have been linked to differences in insular function and structure. Interest in the three interoception sub-constructs and their neural bases has grown with mounting evidence of compromised interoception in numerous mental health and neurodevelopmental disorders (32, 34), including anxiety (44), depression (44), anorexia nervosa (45, 46), and autism spectrum disorder (47–49), to name a select few.

Two lines of evidence motivate research into interoception among CTD populations specifically. First, interoception plays a key role in motor, sensory, and emotional function (30, 31), domains frequently affected in CTDs (50). Second, as noted above, interoception is subserved by the insula, a structure strongly implicated in CTD pathophysiology (51). Enhanced understanding of interoception in CTDs may deepen insight into the phenotypes and neural mechanisms of these disorders.

To date, studies of interoception in CTD have yielded mixed results. Regarding interoceptive accuracy, adults with TS performed less accurately on a heartbeat counting task compared to healthy controls in one study (52) but not another (53). Given concerns that the heartbeat counting method inadequately indexes interoceptive accuracy (54), the latter study also employed a heartbeat discrimination task, finding no group difference between TS and healthy control samples on that task either (53). A pediatric study, also using the heartbeat counting task, identified reduced interoceptive accuracy in children with CTD compared to controls (55). Conflicting findings have similarly emerged from studies of interoceptive sensibility in CTD. Eddy et al. observed heightened interoceptive sensibility, as measured by the Private Body Consciousness Scale (PBCS), in adults with TS relative to controls. Notably, in the TS group neither tic severity nor premonitory urge severity correlated with PBCS score (56). Conversely, Rae et al. found no significant difference between adults with TS and controls in interoceptive sensibility, as measured by the body awareness section of the Body Perception Questionnaire (BPQ) (53), but among TS participants BPQ score did correlate with both tic severity and premonitory urge severity (53). The relationship between interoception and premonitory urge is of particular interest given the insula plays a critical role in emergence of both phenomena (34, 41, 51). Divergent results between studies of interoception in CTD may have arisen from several possible factors, including methodologic differences in assessing interoceptive accuracy or sensibility, disparate eligibility criteria [e.g., Eddy et al. excluded individuals with TS who had psychiatric comorbidities (56) while Rae et al. did not (53)], and relatively small sample sizes [each adult study enrolled between 18 and 21 CTD participants (52, 53, 56); the sole pediatric study enrolled 29 CTD participants (55)]. Furthermore, none of the aforementioned studies adjusted their analyses for the presence or severity of mental health diagnoses that are known to be widespread in CTD populations. The most common comorbid mental health diagnoses among individuals with CTD include attention deficit hyperactivity disorder (ADHD), obsessive-compulsive disorder (OCD), anxiety, and depression, with respective lifetime prevalence rates of 54, 66, 36, and 30%. (57). Many with CTD who do not fulfill formal diagnostic criteria for ADHD, OCD, anxiety, and/or depression still exhibit symptoms of these disorders (58, 59). Each of these comorbid disorders has been associated with abnormal interoceptive accuracy (60–63) and/or sensibility (61, 63–65), and thus, each represents an important potential confound when investigating interoception in CTD. In sum, considerable ambiguity surrounds our understanding of interoception in CTDs.

In the current study, we sought to compare interoceptive sensibility between adults with CTD and healthy controls, adjusting for co-occurring mental health symptoms, and to examine the relationship of interoceptive sensibility with other CTD clinical features, in particular, premonitory urge. To do so, we recruited adults with CTD and sex- and age-matched healthy controls to complete the Multidimensional Assessment of Interoceptive Awareness, Version 2 (MAIA-2), as well as a battery of measures assessing psychiatric symptoms common in CTD populations. CTD participants additionally completed scales quantifying tic severity, premonitory urge severity, and health-related quality of life. We hypothesized the following: first, CTD and control participants would differ in interoceptive sensibility, with CTD participants exhibiting maladaptive interoceptive sensibility, given such a finding in one prior study of adults with TS (56); second, between-group differences in interoceptive sensibility would be partially attributable to between-group differences in co-occurring psychiatric symptom severity, given the known relationship between abnormal interoceptive sensibility and mental health disorders (61, 63–65); and third, after adjusting for other CTD clinical features, premonitory urge severity would positively correlate with interoceptive sensibility, given evidence of a strong correlation between these phenomena in one previous study (53).

## Materials and Methods

### Participants

From February 2021 through February 2022, we recruited adults (≥18 years of age) with CTD and sex- and age-matched adults with no known neurologic or psychiatric diagnoses. English fluency was required for study enrollment. Adults with CTD were recruited from Vanderbilt University Medical Center (VUMC) Tourette Syndrome Clinic and institutional research registries. All CTD participants were interviewed, examined, and diagnosed with a CTD by an experienced movement disorders neurologist (D.I.) using Diagnostic and Statistical Manual of Mental Disorders, 5th edition (DSM-5) criteria. Control participants were recruited via ResearchMatch, a web-based recruitment tool for clinical research (66). Controls completed all study activities online and were not interviewed or examined.

Control participants were one-to-one-matched on sex and age (±5 years) to CTD participants. TS and control participants who completed less than 50% of study measures were excluded from the matching process. All participants were asked to self-report history of any and all of the following conditions: tic disorder, OCD, ADHD, autism spectrum disorder, anxiety, and depression. Controls with a self-reported diagnosis of tic disorder, OCD, ADHD, or autism spectrum disorder were excluded from the matching process, but controls with a history of anxiety and/or depression were included. Data analysis was restricted to matched participants.

Participants provided electronic informed consent and received monetary reimbursement after completing all study activities. This study was approved by the VUMC Institutional Review Board and was conducted in accordance with the Declaration of Helsinki.

### Measures

Table 1 lists the validated measures used in the study. More detailed information on each measure (e.g., number of items, score range, established cut-offs) is available in the Supplementary Material. A movement disorders neurologist (D.I.) administered the Yale Global Tic Severity Scale (YGTSS) (67) to all CTD participants, after which they were emailed unique hyperlinks to the study self-report measures in Research Electronic Data Capture (REDCap). REDCap is a HIPAA-compliant, web-based platform for data collection and storage (68, 69). CTD participants were requested to finish all study measures at their earliest convenience following the YGTSS to minimize time between the clinician-administered and self-report measures. Control participants were emailed unique hyperlinks to the same battery of self-report measures, with the exception that controls did not complete the Premonitory Urge to Tic Scale (PUTS) (70) or the Gilles de la Tourette-Quality of Life Scale (GTS-QOL) (71), both of which are tic disorder-specific. Estimated time to finish the online battery of self-report measures was 30–40 min.

**TABLE 1:** Participant demographic and clinical characteristics.

Variable	Control (*n* = 48)	CTD (*n* = 48)	Wilcoxon rank-sum test for continuous variables
Sex (M: F)	28: 20	28: 20	
Age (years)	31.5 (23.5–49.5)^†^	31 (22–48.5)	*z* = 0.23
**Ethnicity**			
Hispanic or Latino Not Hispanic or Latino Unknown/Not reported	4 43 1	1 46 1	
**Race**			
American Indian or Alaska Native Asian Native Hawaiian or Other Pacific Islander Black or African American White More than one race Unknown/Not reported	0 2 0 6 36 1 3	1 1 0 1 43 1 1	
**Co-occurring conditions, self-reported**			
ADHD OCD Anxiety Depression Autism spectrum disorder	0 0 4 3 0	16 25 27 26 1	
Adult ADHD Self-Report Screening Scale for DSM-5 (ASRS-5)	7.5 (5–9.5)	13 (9.5–16)	*z* = –5.7***
Dimensional Obsessive-Compulsive Scale (DOCS)	9.5 (5–15.5)	15.5 (7.5–28)	*z* = –2.8**
Generalized Anxiety Disorder-7 (GAD-7)	2.5 (0.8–4.5)	9 (2.5–13)	*z* = –4.6***
Patient Health Questionnaire-9 (PHQ-9)	2.5 (1–5)	8 (4.5–15)	*z* = –5.2***
YGTSS Total Tic Score	–	22.5 (15–30)	–
Premonitory Urge to Tic Scale (PUTS)	–	25 (21.5–29)	–
Gilles de la Tourette-Quality of Life Scale (GTS-QOL)	–	31.5 (19.4–51.4)	–

*^†^Median (interquartile range).*

**p < 0.01; ***p < 0.001.

To quantify interoceptive sensibility, we used the Multidimensional Assessment of Interoceptive Awareness, Version 2 (MAIA-2) (72). The MAIA-2 is a 37-item, self-report measure that assesses multiple facets of interoceptive sensibility. Each scale item is a statement to which respondents must select “never” (0) to “always” (5) on a six-point Likert scale. No total MAIA-2 score exists. Rather, individual scale items belong to one of eight MAIA-2 subscales: Noticing (“awareness of uncomfortable, comfortable, and neutral body sensations,” per MAIA-2 developers’ definition), Not-Distracting (“tendency not to ignore or distract oneself from sensations of pain or discomfort”), Not-Worrying (“tendency not to worry or experience emotional distress with sensations of pain or discomfort”), Attention Regulation (“ability to sustain and control attention to body sensations”), Emotional Awareness (“awareness of the connection between body sensations and emotional states”), Self-Regulation (“ability to regulate distress by attention to body sensations”), Body Listening (“active listening to the body for insight”), and Trusting (“experience of one’s body as safe and trustworthy”). For each subscale, higher score signifies more of that construct. The original MAIA was developed via a mixed-methods process, involving concept and item development with an expert panel; focus group testing in instructors of body awareness therapies; cognitive interviewing; and assessment of internal consistency reliability, convergent validity, and incremental validity (73). Due to sub-optimal internal consistency reliability of two subscales of the original MAIA (Not-Worrying and Not-Distracting), the instrument underwent modifications, leading to creation of the MAIA-2 (72). The psychometric properties of the MAIA-2 were evaluated in a large community sample of 1,090 individuals (72). Notably, the MAIA-2 Not-Worrying and Not-Distracting subscales exhibited improved internal consistency reliability relative to the original MAIA versions of these subscales, but their Cronbach’s α values remained slightly below the acceptable cutoff of 0.70 (Noticing 0.64; Not-Worrying 0.67) (72). Despite this limitation, we selected the MAIA-2 for use in the current study because the scale accounts for and differentiates between adaptive and maladaptive dimensions of interoceptive sensibility (74), whereas other scales primarily conceptualize interoceptive sensibility unidimensionally, as anxiety-related somatization (75).

To quantify symptom severity of psychiatric disorders commonly co-occurring with CTDs, all participants completed the following validated self-report measures: Adult ADHD Self-Report Screening Scale for DSM-5 (ASRS-5) (76), Dimensional Obsessive-Compulsive Scale (DOCS) (77), Generalized Anxiety Disorder-7 (GAD-7) (78), and Patient Health Questionnaire-9 (PHQ-9) (79). CTD participants were also administered the YGTSS, as noted above, as well as the PUTS and GTS-QOL.

### Statistical Approach

To provide non-parametric measures of central tendency and dispersion for continuous variables, we calculated medians and interquartile ranges. Missing item responses were imputed from mean, non-missing responses of all matched participants.

To examine internal consistency reliability of the MAIA-2 in the current sample, we computed McDonald’s ω for each of the eight subscales across all participants. McDonald’s ω is an estimate of internal consistency reliability that is robust when the assumption of τ-equivalence is violated and is thus more appropriate than Cronbach’s α for most psychological self-report measures (80).

To examine between-group differences in interoceptive sensibility, we contrasted CTD and control group scores on each of the eight MAIA-2 subscales with the Wilcoxon-rank sum test. To account for multiple comparisons, we employed the false discovery rate-controlling procedure developed by Benjamini et al. (81). The magnitude of the Wilcoxon rank-sum test statistic functions as a non-parametric measure of effect size (82).

For MAIA-2 subscales with significantly different scores between the groups, we conducted secondary analyses to assess the effect of co-occurring psychiatric symptoms on the association between the MAIA-2 subscale and CTD diagnosis. To do so, we constructed multivariable linear regression models with the given MAIA-2 subscale as the dependent variable and the following as independent variables: sex, age, CTD diagnosis, ASRS-5 score, DOCS score, and GAD-7 score. PHQ-9 score was not included as an independent variable due to its strong correlation with GAD-7 score in both CTD (*r*_*s*_ = 0.66) and control (*r*_*s*_ = 0.73) groups. We next constructed a reduced model for the given MAIA-2 subscale, with the same set of independent variables except CTD diagnosis was removed. For each regression model, we plotted histograms of residuals to visually inspect for deviations from normality, plotted residuals against the independent variable to visually inspect for heteroskedasticity, calculated the Breusch–Pagan test statistic to quantify heteroskedasticity, calculated the variance inflation factor (VIF) for independent variables to identify significant multicollinearity (pre-specified as VIF > 5) (83), and performed a regression specification error test to assess for likelihood of omitted variables. Adjusted *R*^2^ indexed model goodness-of-fit. Likelihood ratio tests and Akaike information criteria (AIC) were used to compare goodness-of-fit between full and reduced models. We conducted *post hoc t*-tests of the full models to examine the association between independent and dependent variables, with a pre-specified significance threshold of *p* < 0.05.

As an exploratory analysis, we contrasted MAIA-2 subscale scores between the subset of CTD participants with no reported ADHD or OCD and their sex- and age-matched controls. This analysis was performed to facilitate results comparison with other studies in which individuals with CTD were excluded for comorbid diagnoses of ADHD or OCD (56). We applied Benjamini et al’s false discovery rate-controlling procedure to account for multiple comparisons (81). Of note, all other analyses discussed in the Methods section were conducted with data from the entire CTD cohort; only this exploratory analysis was conducted with data from a subset of the cohort.

To assess the interrelationship between measures within each participant group, we calculated Spearman’s rank correlations (*r*_*s*_) between scale scores, using the aforementioned false discovery rate-controlling procedure to account for multiple comparisons (81).

To further examine the association of interoceptive sensibility with premonitory urge in the CTD sample, we constructed a multivariable linear regression model with PUTS score as the dependent variable. Given our sample size, we were insufficiently powered to incorporate all eight MAIA-2 subscales into the model. We thus first sought to reduce the dimensionality of the MAIA-2 scale using hierarchical cluster analysis, with average linkage, on subscale scores from CTD participants. Prior to clustering, MAIA-2 subscale scores were standardized. A dissimilarity matrix, with the eight subscales as individual variables, was constructed using Euclidian distance as the metric. Based upon the dendrogram yielded by the cluster analysis of the MAIA-2 subscales, we identified a three-variable-cluster solution. For the premonitory urge regression model, the following served as independent variables: the three-variable solution to the MAIA-2 hierarchical cluster analysis, DOCS score, and YGTSS Total Tic Score. DOCS score and YGTSS Total Tic Score were selected as model covariates given the established association of premonitory urge severity with severity of obsessive-compulsive symptoms and tics (84–86). Study sample size precluded addition of other clinical variables and interaction terms into the premonitory urge regression model. We employed the same regression diagnostics outlined earlier in the Methods section.

Statistical analyses were conducted in STATA 15.0 and Excel 16.5.

## Results

### Population

Forty-eight participants with CTD (46 with TS, 2 with chronic motor tic disorder) and 68 control participants completed more than 50% of study measures. From the pool of control participants, four were excluded due to self-reported history of ADHD (*n* = 1), OCD (*n* = 2), or both (*n* = 1). From this remaining pool, 48 control participants were one-to-one sex- and age-matched to CTD participants. All subsequent analyses refer to matched participants. Data from the final cohort were > 99.9% complete, with missing responses only from single items of the PHQ-9 (for one participant) and GAD-7 (for two other participants). CTD participants completed self-report measures a median of 1 day (interquartile range 0 –9.5 days) following YGTSS administration.

Table 1 contains demographic and clinical information for the matched sample. Age-matching was successful, with no significant difference in age between groups. The sample as a whole was predominantly non-Hispanic white, though the control population was slightly more diverse. Adults with CTD endorsed significantly more severe symptoms of ADHD, OCD, anxiety, and depression relative to controls.

Internal reliability consistency for all eight MAIA-2 subscales, across the entire study population, was above the conventional threshold of 0.70, with McDonald’s ω ranging from 0.74 –0.93. McDonald’s ω for each MAIA-2 subscale is provided in the Supplementary Material.

### Between-Group Contrasts of MAIA-2 Subscale Scores

After controlling for the false discovery rate, CTD and control participant scores differed for two MAIA-2 subscales: Noticing and Not-Worrying (see Table 2). CTD participants were 65.8% (95% CI: 54.6–76.9%) more likely to have a higher MAIA-2 Noticing score than controls, while controls were 67.7% (95% CI: 56.8–78.5%) more likely to have a higher MAIA-2 Not-Worrying score than CTD participants. Respectively, findings suggest adults with CTD experience increased awareness of bodily sensations in general, as well as heightened worry in response to uncomfortable bodily sensations. Between-group difference for the MAIA-2 Trusting subscale approached significance (*p* = 0.046), but significance did not survive correction for multiple comparisons.

**TABLE 2:** Between-group contrasts for MAIA-2 subscale scores.

MAIA-2 subscale	Control (*n* = 48)	CTD (*n* = 48)	Wilcoxon rank-sum test
Noticing	2.1 (1.0–3.0)^†^	3.0 (2.1–3.5)	*z* = –2.7*
Not-Distracting	2.8 (2.0–3.8)	2.7 (1.6–3.5)	*z* = 1.3
Not-Worrying	3.1 (2.6–3.8)	2.8 (1.8–3.2)	*z* = 3.0*
Attention Regulation	2.7 (1.8–3.1)	1.9 (1.4–3.1)	*z* = 1.2
Emotional Awareness	2.6 (1.7–3.2)	2.8 (2.1–3.6)	*z* = –1.0
Self-Regulation	2.8 (1.5–3.6)	2.1 (1.3–3.0)	*z* = 1.6
Body Listening	1.5 (0.7–3.0)	1.3 (0.8–2.0)	*z* = 0.6
Trusting	3.0 (2.7–4.0)	2.7 (2.0–3.8)	*z* = 2.0

*^†^Median (interquartile range).*

*Significant at p < 0.019 (threshold as determined by false discovery rate-controlling procedure).

Results of the multivariable linear regression analysis for the MAIA-2 Noticing and Not-Worrying subscales are shown in Table 3. Full and reduced models for these subscales satisfied the assumptions of multivariable linear regression, as assessed by the diagnostic procedures outlined in the Methods. The Supplementary Material contains histograms of the model residuals. For both the Noticing and the Not-Worrying subscales, the full models explained a statistically significant portion of the subscale score variance. However, the full model for the Noticing subscale explained a relatively low percentage of the score variance (adj *R*^2^ = 0.09), and none of the selected independent variables were significantly associated with the subscale score. Adjusted *R*^2^ and AIC values for the Noticing subscale full model were similar to those of the reduced model, and the goodness-of-fit did not significantly differ between these models, as determined by the likelihood ratio test, suggesting that CTD diagnosis did not significantly contribute to the Noticing subscale model goodness-of-fit. The full model for the Not-Worrying subscale explained a moderate percentage of the score variance (adj *R*^2^ = 0.30), and sex and DOCS total score were significantly associated with the Not-Worrying subscale score, while CTD diagnosis was not. These findings indicate female sex and more severe obsessive-compulsive symptoms were associated with greater tendency to worry about uncomfortable bodily sensations. As with the Noticing subscale models, adjusted *R*^2^ and AIC values for the full Not-Worrying models were similar to those of the reduced model, and the likelihood ratio test statistic from comparison of these models did not reach significance, suggesting that CTD diagnosis did not significantly contribute to the Not-Worrying subscale model goodness-of-fit.

**TABLE 3:** Regression model diagnostics and results for MAIA-2 Noticing and Not-Worrying Subscales.

Dependent variable^§^		Independent variables	VIF^†^	Breusch–Pagan test^‡^	Specification error test^∧^	Independent variables significantly associated with dependent variable	Model goodness-of -fit indices	Likelihood ratio
	Full							
		CTD diagnosis Age Sex GAD-7 score DOCS score ASRS-5 score	1.74 1.23 1.19 2.17 1.78 1.89	χ^2^(1) = 0.07 *p* = 0.80	F(3,86) = 2.18 *p* = 0.10	–	F(6,89) = 2.54 *p* < 0.05 *R*^2^ = 0.15 adj *R*^2^ = 0.09 AIC^¶^ = 322.6	
MAIA-2 Noticing subscale score								χ^2^(1) = 0.77 *p* = 0.38
	Reduced							
		Age Sex GAD-7 score DOCS score ASRS-5 score	1.19 1.18 1.97 1.78 1.51	χ^2^(1) = 0.01 *p* = 0.92	F(3,87) = 2.16 *p* = 0.10	–	F(5,90) = 2.92 *p* < 0.05 *R*^2^ = 0.14 adj *R*^2^ = 0.09 AIC^¶^ = 321.4	

	Full							
		CTD diagnosis Age Sex GAD-7 score DOCS score ASRS-5 score	1.74 1.23 1.19 2.17 1.78 1.89	χ^2^(1) = 0.25 *p* = 0.62	F(3,86) = 0.75 *p* = 0.52	Sex: β = 0.42 (95% CI: 0.06–0.78) *t* = 2.3, *p* < 0.05 DOCS score: β = –0.028 (95% CI: –0.05 - –0.01) *t* = –3.1, *p* < 0.01	F(6,89) = 7.77 *p* < 0.0001 *R*^2^ = 0.34 adj *R*^2^ = 0.30 AIC = 238.1	
MAIA-2 Not-Worrying subscale score								χ^2^(1) = 2.99 *p* = 0.08
	Reduced							
		Age Sex GAD-7 score DOCS score ASRS-5 score	1.19 1.18 1.97 1.78 1.51	χ^2^(1) = 0.29 *p* = 0.59	F(3,87) = 0.57 *p* = 0.64	Sex: β = 0.40 (95% CI: 0.03–0.76) *t* = 2.2, *p* < 0.05 DOCS score: β = –0.028 (95% CI: 0.05 - –0.01) *t* = –3.0, *p* < 0.01	F(5,90) = 8.59 *p* < 0.0001 *R*^2^ = 0.32 adj *R*^2^ = 0.29 AIC = 239.1	

*^§^Diagnostics and results are stratified into the full and reduced regression models, as noted by vertical text in the rightmost portion of this column.*

*^†^VIF, variance inflation factor.*

*^‡^p< 0.05 for Breusch–Pagan test indicates significant likelihood of heteroskedasticity.*

*∧p < 0.05 for regression specification error test indicates significant likelihood the model has omitted variables.*

*^¶^AIC, Akaike information criteria.*

Fifteen CTD participants reported no history of ADHD or OCD. The Supplementary Material contains full results from the comparison of MAIA-2 subscale scores and other scale scores between this CTD subset and their matched controls. Even within this subset, CTD participants exhibited more severe symptoms of ADHD, anxiety, and depression (see Supplementary Material). After correcting for multiple comparisons, group scores did not significantly differ for any of the scales. However, CTD participants without reported OCD or ADHD trended toward lower Self-Regulation subscale score (*z* = 2.2, *p* = 0.03) and higher Not-Worrying subscale score (*z* = 1.7, *p* = 0.09).

### Clinical Correlates of MAIA-2 Subscale Scores

**FIGURE 1:**
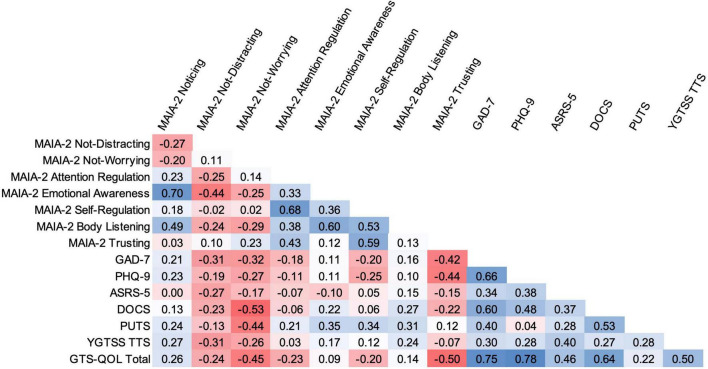
Bivariate correlation matrix for CTD participants. Intensity of shading reflects magnitude of Spearman rank correlation, with blue indicating positive correlation and red indicating negative correlation. Correlations with absolute values ≥ 0.34 are significant, as per the false discovery rate-controlling procedure. TTS, Total Tic Score.

Across the entire CTD participant group, select MAIA-2 subscale scores significantly correlated with scores from several other measures (see Figure 1). MAIA-2 Not-Worrying score negatively correlated with DOCS (*r*_*s*_ = –0.53, *p* < 0.001), PUTS (*r*_*s*_ = –0.44, *p* < 0.01), and GTS-QOL (*r*_*s*_ = –0.45, *p* < 0.01) scores, indicating that higher Not-Worrying scores were associated with lower obsessive-compulsive symptom severity, lower premonitory urge severity, and higher health-related quality of life. MAIA-2 Trusting score negatively correlated with GAD-7 (*r*_*s*_ = –0.42, *p* < 0.01), PHQ-9 (*r*_*s*_ = –0.44, *p* < 0.01), and GTS-QOL (*r*_*s*_ = –0.50, *p* < 0.001) scores, indicating that higher Trusting scores were associated with less anxiety, less depression, and higher health-related quality of life. In addition to MAIA-2 Not-Worrying score, PUTS score significantly correlated with MAIA-2 Emotional Awareness (*r*_*s*_ = 0.35, *p* < 0.05) and Self-Regulation (*r*_*s*_ = 0.34, *p* < 0.05) scores. GAD-7 and PHQ-9 were the measures most strongly correlated with GTS-QOL (*r*_*s*_ = 0.75, *p* < 0.0001 and *r*_*s*_ = 0.78, *p* < 0.0001, respectively). PUTS score did not significantly correlate with YGTSS Total Tic Score after correction for multiple comparisons (*r*_*s*_ = 0.28, *p* = 0.05). Notably, the degree of correlation between PUTS score and YGTSS Total Tic Score in our sample closely aligned with results from a recent meta-analysis examining the relationship between severity of premonitory urges and tics (84). The correlation matrix for control participants is available in the Supplementary Material.

**FIGURE 2:**
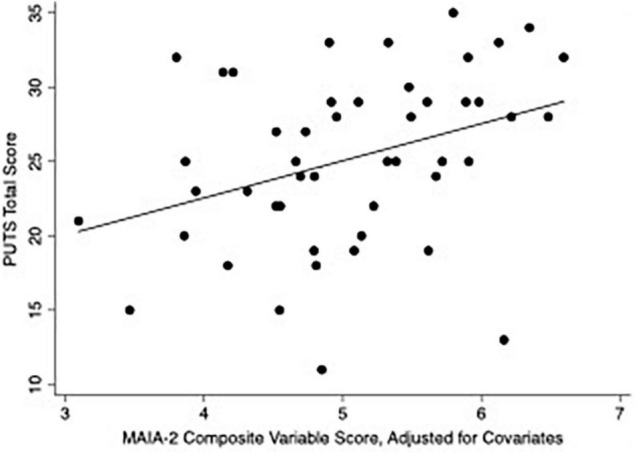
Scatterplot of PUTS score versus MAIA-2 composite variable score. The MAIA-2 composite variable score is the mean of the scores from the following six MAIA-2 subscales: Noticing, Attention Regulation, Emotional Awareness, Self-Regulation, Body Listening, and Trusting. for the above plot, the composite score was adjusted for MAIA-2 Not-Worrying score, MAIA-2 Not-Distracting score, DOCS score, and YGTSS Total Tic Score, by inserting CTD-group median values for these variables into the regression model.

In the hierarchical cluster analysis of MAIA-2 subscales within the CTD group, Not-Worrying and Not-Distracting were most dissimilar from the other six subscales (see dendrogram in Supplementary Material), in accord with several other studies (87–89). We thus generated a composite variable of the other six subscales (Noticing, Attention Regulation, Emotional Awareness, Self-Regulation, Body Listening, and Trusting) by averaging their scores. We then constructed a multivariable linear regression model with PUTS score as the dependent variable and the following as independent variables: MAIA-2 Not-Worrying score, MAIA-2 Not-Distracting score, MAIA-2 composite variable score (i.e., mean score from the six MAIA-2 subscales besides Not-Worrying and Not-Distracting), DOCS score, and YGTSS Total Tic Score (see Table 4). The model satisfied multivariable linear regression assumptions. The residuals histogram is provided in the Supplementary Material. Under this regression model, the MAIA-2 composite variable score, DOCS score, and YGTSS Total Tic Score were each independently associated with PUTS score. Figure 2 plots PUTS score against the MAIA-2 composite variable score.

**TABLE 4:** Regression model for PUTS.

Dependent variable	Independent variables	VIF^†^	Breusch–Pagan Test	Specification error test	Model goodness-of-fit indices	Independent variables significantly associated with dependent variable
PUTS score	MAIA-2 Not-Worrying score MAIA-2 Not-Distracting score MAIA-2 composite variable score^§^ DOCS score YGTSS Total Tic score	1.55 1.43 1.14 1.53 1.34	χ^2^(1) = 0.19 *p* = 0.66	F(3,39) = 2.79 *p* = 0.053	F(5,42) = 7.03 *p* < 0.0001 *R*^2^ = 0.46 adj *R*^2^ = 0.39 AIC^¶^ = 287.0	MAIA-2 composite variable score: β = 2.52 (95% CI: 0.79–4.24) *t* = 2.95, *p* < 0.01 DOCS score: β = 0.14 (95% CI: 0.02–0.25) *t* = 2.42, *p* < 0.05 YGTSS TTS: β = 0.08 (95% CI: 0.00–0.16) *t* = 2.02, *p* < 0.05

*^§^MAIA-2 composite variable = mean of MAIA-2 Noticing, Attention Regulation, Emotional Awareness, Self-Regulation, Body Listening, and Trusting Subscale.*

*^†^VIF, variance inflation factor.*

*^¶^AIC, Akaike information criteria.*

## Discussion

In this study of interoceptive sensibility in adults with CTD, we identified three novel findings. First, only select dimensions of interoceptive sensibility (Noticing and Not-Worrying) differ between adults with CTD and healthy controls. Second, anxiety-associated aspects of interoceptive sensibility are more strongly associated with female sex and obsessive-compulsive symptom severity than with CTD diagnosis. Third, premonitory urge severity is significantly associated with interoceptive sensibility, even after controlling for severity of tics and obsessive-compulsive symptoms. We will discuss these findings sequentially.

In the current study, CTD participants endorsed a greater tendency to worry about sensations of bodily discomfort, as evidenced by their significantly lower MAIA-2 Not-Worrying subscale scores relative to controls. Two prior studies in adults with CTD used alternate self-report measures to quantify interoceptive sensibility: the Private Body Consciousness Scale (PBCS) (56) and the body awareness section of the Body Perception Questionnaire (BPQ) (53). The PBCS and the BPQ predominantly index a disposition to anxiety-associated somatization (48, 75, 90), similar to the MAIA-2 Not-Worrying subscale (41, 87, 88). Findings from these previous studies of interoceptive sensibility in CTD were discrepant: Eddy et al. identified increased interoceptive sensibility (as measured by the PBCS) in CTD participants relative to controls (56), whereas Rae et al. did not identify such a between-group difference (with the BPQ) (53). Importantly, neither of these studies incorporated sex or severity of co-occurring psychiatric symptoms into their analyses (53, 56). These clinical factors are critical considerations in studies of interoceptive sensibility given evidence of divergent interoceptive sensibility between the sexes (65, 91) and atypical interoceptive sensibility in depressive (92, 93), anxiety (94, 95), and obsessive-compulsive disorders (64). Among individuals with CTD, lifetime prevalence of depression, anxiety, and OCD is 30, 36, and 66%, respectively (57), highlighting the relevance of these conditions when researching interoception in CTD populations. In our sample, after adjusting for covariates, the MAIA-2 Not-Worrying subscale score was independently associated with sex and severity of obsessive-compulsive symptoms, but not with CTD diagnosis. Additionally, CTD diagnosis did not significantly contribute to model goodness-of-fit. Collectively, these findings suggest that observed between-group differences in anxiety-associated somatization were more attributable to obsessive-compulsive symptoms (since groups were sex-matched) than to CTD diagnosis. Female participants and participants with more severe obsessive-compulsive symptoms reported a greater tendency to worry about uncomfortable bodily sensations. These findings align with results from studies in non-tic disorder populations. In a large community sample (*n* = 367), women and men displayed distinct interoceptive sensibility profiles, with women tending to more frequently attend to bodily sensations, relate emotional state with bodily sensations, and experience distress with uncomfortable bodily sensations (96). Sex differences in interoceptive sensibility and accuracy are posited (91) to contribute to sex-specific vulnerabilities (91), symptom profiles (91, 97), and treatment responses (65) in anxiety and depression. Previous studies have revealed that individuals with OCD also exhibit heightened worry about uncomfortable bodily sensations, as well as greater tendency to distract themselves from bodily sensations and to experience the body as untrustworthy (64). In conjunction with this prior research, the current study findings suggest the relationships of sex and obsessive-compulsive symptoms with the anxiety-associated dimension of interoceptive sensibility are transdiagnostic. Results underscore the need to assess and adjust for sex and common co-occurring psychiatric symptoms when examining interoceptive sensibility in CTD.

Adults with CTD in the current study also reported an enhanced general awareness of bodily sensations, as reflected in their higher MAIA-2 Noticing subscale scores, compared to healthy controls. However, in the regression analysis, CTD diagnosis, severity of co-occurring psychiatric symptoms, and sex collectively explained a low percentage of the Noticing subscale score variance, and none of these variables were significantly associated with the subscale score. Furthermore, MAIA-2 Noticing subscale score did not significantly correlate with scores of any non-MAIA-2 measures in CTD or control participants. While all other MAIA-2 subscales assess an adaptive dimension of interoceptive sensibility, the Noticing subscale indexes a neutral dimension (74), with questions such as “I notice changes in my breathing, such as whether it slows down or speeds up.” Notably, individuals with OCD also have elevated scores on this subscale (64). Further research with larger sample sizes may help to clarify the relationship of this Noticing dimension of interoceptive sensibility to other facets of the CTD phenotype.

CTD and control participants in our study did not significantly differ on any other MAIA-2 subscales. Between-group differences in the Trusting subscale scores approached significance (*p* = 0.046), with the CTD group more likely to experience the body as untrustworthy. The increased tendency to distrust (lower Trusting subscale score) and to worry about bodily discomfort (lower Not-Worrying subscale score) are consistent with a maladaptive interoceptive sensibility profile. Dimensional profiles of interoceptive sensibility vary across mental health (64, 98–100) and pain (101, 102) disorders, and individual dimensions appear to have prognostic value in certain settings (65, 100, 103). Future studies of interoceptive sensibility in CTD should account for the multidimensionality of this construct.

The relationship between interoceptive sensibility and premonitory urge in CTD is of considerable interest given the phenomenological overlap and shared neural underpinnings (as will be discussed below) between these phenomena. In our sample, after controlling for severity of tics and obsessive-compulsive symptoms, premonitory urge was significantly associated with the composite of MAIA-2 Noticing, Attention Regulation, Emotional Awareness, Self-Regulation, Body Listening, and Trusting subscales. Higher score on this MAIA-2 composite variable was associated with more severe premonitory urge. The six subscales comprising this MAIA-2 composite variable have collectively been labeled a general measure of body awareness since, as a group, they reflect perception of bodily “changes and rhythms” rather than of bodily response to negative emotions (87). In contrast, the MAIA-2 Not-Worrying and Not-Distracting subscales focus on reactions to bodily pain and discomfort (87). The MAIA-2 Not-Worrying subscale, in particular, correlates closely with anxiety measures (41, 87, 88). Prior studies examining the association between premonitory urge and interoceptive sensibility in CTD have used measures of interoceptive sensibility that primarily assess anxiety-associated somatization: Rae et al. (using the BPQ) observed a significant correlation between urge and interoceptive sensibility (53), while Eddy et al. (using the PBCS) did not (56). Neither study accounted for co-occurring psychiatric diagnoses or symptoms in their analyses. It is notable that in the current study, premonitory urge severity correlated more strongly with the MAIA-2 Not-Worrying subscale (*r*_*s*_ = –0.44) than with the other MAIA-2 subscales. However, after controlling for the multiple dimensions of interoceptive sensibility, as well as for severity of obsessive-compulsive symptoms and tics, the general measure of body awareness was significantly associated with premonitory urge severity, while the Not-Worrying subscale was not.

The above finding has potential therapeutic implications. Premonitory urges are experienced by 80–90% of adolescents and adults with CTD and are more distressing than tics for many patients (51). Premonitory urges also serve as an integral component of comprehensive behavioral intervention for tics (CBIT), an evidenced-based therapy for tics with a treatment effect size similar to approved medications (104) and sustained benefit for at least six months post-treatment (105). During CBIT, patients are first trained to self-monitor for a specific tic and its associated premonitory urge (104). Patients then learn to implement a volitional movement physically incompatible with the tic (a so-called competing response) when the premonitory urge is detected. Given CBIT’s operational reliance on premonitory urge, one might speculate that severity of urges would portend better response to this intervention. However, in a pooled analysis of adults and children with CTD (*n* = 248), baseline severity of premonitory urges predicted less improvement with CBIT (106). In that same analysis, severity of premonitory urges failed to improve at the end of the 10-week CBIT treatment period, even though tic severity significantly decreased (106). This and other evidence (107, 108) demonstrate that severity of tics and premonitory urges are dissociable, and in fact, decoupling of the premonitory-urge tic complex is one mechanism by which CBIT is postulated to exert its effect (105). Ultimately, the presence of the premonitory urge itself may be less clinically important than the valence attached to the urge. Under this theoretical framework, a key function of CBIT is to facilitate re-appraisal of premonitory urges as non-threatening phenomena that permit adaptive behaviors. More generally, re-tuning conscious and subconscious responses to somatic sensations is commonly employed in numerous behavioral interventions across various disorders. For example, addition of interoceptive training to standard therapies for anxiety disorders (109, 110), eating disorders (111), and select pain disorders (112, 113) yields incremental benefit in mitigating symptoms, demonstrating the transdiagnostic utility of such an approach. A more refined understanding of the relationship between interoceptive sensibility and premonitory urge may allow further optimization of behavioral therapies for CTD.

The current study assessed interoceptive sensibility, but interoceptive accuracy is also aberrant in CTDs. Both adults (52) and children (55) with CTD exhibit reduced interoceptive accuracy, as gauged by a heartbeat counting task. Notably, another study comparing adults with CTD and healthy controls did not identify between-group differences in a heartbeat counting task or a heartbeat discrimination task, but the investigators did observe that the discrepancy between interoceptive accuracy and interoceptive sensibility (so-called trait interoceptive predictive error) was significantly greater in CTD participants (53). This discordance between interoceptive accuracy and interoceptive sensibility suggests these individuals experience heightened subjective responses to bodily signals but exhibit a diminished ability to objectively detect those signals (53). High trait interoceptive predictive error is also evident in individuals with autism spectrum disorder (49, 114) and anxiety (49). Some investigators propose, under a Bayesian predictive coding framework, that the mismatch between interoceptive accuracy and interoceptive sensibility is more relevant than either phenomenon considered in isolation (49, 115, 116).

Both interoceptive accuracy (34, 41, 42) and interoceptive sensibility (41) are subserved by the insula, a structure strongly implicated in CTD pathophysiology as well (51). The insula is functionally segregated into posterior, ventral anterior, and dorsal anterior subdivisions (61). Bottom-up interoceptive signals from the body are received in the posterior insula and there integrated with exteroceptive and proprioceptive inputs (34). This information is then relayed to the ventral anterior and dorsal anterior insula where it is assimilated with top-down emotional and cognitive input from other cortical and sub-cortical structures, yielding a complex, topographically-organized representation of the bodily state contingent on physiology, affect, and prior beliefs (30, 34, 61). In accord with this empirically grounded model, individual differences in insular structure and function predict interoceptive accuracy and interoceptive sensibility. Enhanced hemodynamic activity in the right insula predicts healthy individuals’ accuracy in a heartbeat detection task (42), and increased gray matter volume in the same region correlates with increased task accuracy and increased subjective awareness of bodily sensations (42). Maladaptive dimensions of interoceptive sensibility (specifically, decreased attentional control and increased distraction and worry) are associated with increased hemodynamic activity in a distributed network involving the insula, somatosensory cortex, motor cortex, and cingulate cortex (41). Given the critical role of the insula in subserving interoception and given the altered interoception in CTDs, it is unsurprising that abnormalities in insular structure and function have been observed in CTD populations. In TS, the insula exhibits reduced cortical thickness (117), reduced GABA_*A*_ receptor binding (27), and enhanced functional connectivity with frontal and striatal regions (118). Of the clinical manifestations of CTD, the insula is most clearly linked with premonitory urge. Severity of premonitory urges correlates with left insula cortical thickness (117) and with extent of functional connectivity between the right insula and the bilateral supplementary motor areas (118). In the one to two seconds preceding a tic, when premonitory urges are subjectively experienced, a diffuse cortical network involving the insula activates (119, 120). These tic disorder-specific findings align with the wider literature demonstrating that the insula subserves urge-to-action (51) and provides essential input to inform movement (30). Future research is needed to explore the relationship of insular structure and function to interoception anomalies in CTDs.

Additionally, given evidence of interoception abnormalities in many neurodevelopmental and mental health disorders, cross-disorder comparisons of interoception are of prime interest. In particular, research directly comparing interoception between CTD and OCD populations would significantly advance insight into the transdiagnostic impact of altered interoception. As discussed previously, one dimension of interoceptive sensibility, anxiety-associated somatization, is prevalent in both CTD and OCD samples (56, 61, 64). Furthermore, among both CTD and OCD populations, many individuals experience “not just right” sensations (51, 64). In CTD, such sensations manifest as premonitory urges with this specific quality (51), while in OCD, the sensations occur in the context of repetitive behaviors (64). Severity of “not just right” sensations in OCD correlates with overall tendency to notice bodily sensations (as indexed by the MAIA Noticing subscale) (64). Recent translational work showed that distinct facets of interoceptive sensibility in OCD are differentially associated with insula functional connectivity (121). Cross-disorder investigations promise to further elucidate the neural mechanisms underpinning the sensory dysfunction evident in CTD and OCD.

Our study has several notable limitations. First, while our sample size was larger than previous studies examining interoceptive sensibility in CTD, we may have been under-powered to detect possible between-group differences across all dimensions of interoceptive sensibility. Second, due to the study sample size and number of variables under consideration, our regression analyses did not incorporate interaction or medication terms. Third, co-occurring psychiatric symptoms were quantified with self-report scales rather than gold-standard clinician-administered measures, though the scales employed demonstrate good convergent validity with clinician-administered instruments (76–79). Last, the majority of CTD participants were recruited from a tertiary care clinic, and across both CTD and control groups, participants were predominantly white and non-Hispanic. Both of these issues undermine generalizability of the study findings to the broader, diverse CTD population. The relevance of study findings to the pediatric CTD population is also unclear. One study has examined interoceptive accuracy in children with CTD (55), but to our knowledge, no studies have assessed interoceptive sensibility in this population, precluding results comparison between pediatric and adult CTD samples.

Despite the above limitations, study results revealed three novel findings: adults with CTD experience increased anxiety-associated somatization and increased general body awareness relative to healthy controls; anxiety-associated somatization is more closely associated with sex and obsessive-compulsive symptoms than with CTD diagnosis; and increased general body awareness is associated with greater severity of premonitory urges. Future research is warranted to determine the therapeutic relevance of interoceptive sensibility for CTDs and to clarify the translational links between interoceptive sensibility, interoceptive accuracy, and CTD neurobiology.

## Data Availability Statement

The raw, de-identified data supporting the conclusions of this article will be made available by the authors, without undue reservation.

## Ethics Statement

The studies involving human participants were reviewed and approved by Vanderbilt Human Research Protections Program. The patients/participants provided their written informed consent to participate in this study.

## Author Contributions

AN and DI conceived and designed the study, with assistance from CC. ME and DI implemented the study protocol and collected the data. AN and DI performed the statistical analysis and drafted the initial manuscript. ME, HR, and CC critically reviewed and revised the manuscript. All authors approved the submitted version of the manuscript.

## Conflict of Interest

The authors declare that the research was conducted in the absence of any commercial or financial relationships that could be construed as a potential conflict of interest.

## Publisher’s Note

All claims expressed in this article are solely those of the authors and do not necessarily represent those of their affiliated organizations, or those of the publisher, the editors and the reviewers. Any product that may be evaluated in this article, or claim that may be made by its manufacturer, is not guaranteed or endorsed by the publisher.
