# Supplementary material for: Altered Interoceptive Sensibility in Adults With Chronic Tic Disorder
Source: Front Psychiatry. 2022 Jun 21;13:914897. doi: 10.3389/fpsyt.2022.914897 (PMC9253400; doi:10.3389/fpsyt.2022.914897)
Supplement: Supplementary file 1 [file Table_1.DOCX]

Supplementary Material

## Supplementary Tables

**Table 1.** Study Measure Properties and Psychometric Details

| Scale | Self-Report vs Clinician-Administered | Number of Items | Item Format | Time Frame | Example Items | Scale Range | Scoring | Subscales | Validation References |
| --- | --- | --- | --- | --- | --- | --- | --- | --- | --- |
| Multidimensional Assessment of Interoceptive Awareness, Version 2 (MAIA-2)(1) | Self-report | 37 | Statement to which respondents must select one answer from a 6-point Likert scale, ranging from “Never” (0) to “Always” (5) | None explicitly provided | “When I am tense I notice where the tension is located in my body.”  “I ignore physical tension or discomfort until they become more severe.” | No total scale score exists. Rather, individual scale items belong to one of 8 sub-scales (see other column). | Higher score on each subscale (see next column) indicates more of that construct. No established severity cut-offs. | Noticing,  Not-Distracting,  Not-Worrying,  Attention Regulation,  Emotional Awareness,  Self Regulation,  Body Listening,  Trusting | (1) |
| Adult ADHD Self-Report Screening Scale for DSM-5 (ASRS-5)(2) | Self-report | 6 | Statement to which respondents must select one answer from a 5-point Likert scale, ranging from “Never” (0) to “Very often” (4) | Past 6 months | “How often do you have difficulty concentrating on what people are saying to you even when they are speaking to you directly?” | 0-24 | Higher score indicates more ADHD symptoms. In clinical samples, ASRS-V score ≥ 14 was 81% sensitive and 70% specific for detecting ADHD.(2) | - | (2) |
| Dimensional Obsessive-Compulsive Scale (DOCS)(3) | Self-report | 20 | Multiple choice question to which respondents must select one of 5 options | Past 1 month | “About how much time have you spent each day thinking about contamination and engaging in washing or cleaning behaviors because of contamination?” | 0-80 | Higher score indicates more obsessive-compulsive symptoms.  In clinical samples, DOCS score ≥ 21 was 70% sensitive and 70% specific in discriminating OCD from other anxiety disorders.(3) | Contamination,  Harm,  Unacceptable Thoughts,  Symmetry | (3) |
| Generalized Anxiety Disorder-7 (GAD-7)(4) | Self-report | 7 | Statement to which respondents must select one answer from a 4-point Likert scale, ranging from “Not at all” (0) to “Nearly every day” (3) | Past 2 weeks | “Over the last 2 weeks, how often have you been bothered by the following problems?  Feeling nervous, anxious, or on edge” | 0-21 | Higher score indicates more anxiety. A score ≥ 10 has acceptable sensitivity and specificity for detecting generalized anxiety disorder. (4) | - | (4,5) |
| Patient Health Questionnaire-9 (PHQ-9)(6) | Self-report | 9 | Statement to which respondents must select one answer from a 4-point Likert scale, ranging from “Not at all” (0) to “Nearly every day” (3) | Past 2 weeks | “Over the last 2 week how often have you been bothered by any of the following problems?  Little interest or pleasure in doing things” | 0-27 | Higher score indicates more depression. A meta-analysis found that cut-off scores between 8 and 11 had acceptable sensitivity and specificity for detecting major depressive disorder.(7) | - | (8) |
| Yale Global Tic Severity Scale (YGTSS)(9) Total Tic Score | Clinician-administered | 10 | Following a tic inventory, clinician conducts a semi-structured interview to determine severity of motor and vocal tics (separately) on 5 domains: number, frequency, intensity, complexity, and interference, after which a rating of 0 (least severe) to 5 (most severe) is assigned to each of these domains | Past 1 week | - | 0-50 | Higher score indicates more severe tics. No established severity cut-offs. | Motor Tic Score,  Vocal Tic Score | (10) |
| Premonitory Urge to Tic Scale (PUTS)(11) | Self-report | 10 | Statement to which respondents must select one answer from a 4-point Likert scale, ranging from “Not at all” (1) to “Very much” (4) | None explicitly provided | “Right before I do a tic I feel like my insides are itchy” | 9-36 | Higher score indicates more severe premonitory urge. No established severity cut-offs. | - | (12) |
| Gilles de la Tourette-Quality of Life Scale (GTS-QOL)(13) | Self-report | 27 | Statement to which respondents must select one answer from a 5-point Likert scale, ranging from “No problem” (0) to “Extreme problem” (4) | Past 4 weeks | “In the last 4 weeks have you...  Been unable to control all your movements?” | 0-100 | Higher score indicates worse health-related quality of life. No established severity cut-offs. | Psychological, Physical/Activities of Daily Living, Obsessive-Compulsive, Cognitive | (13) |

References for Supplementary Material Table 1

[1] Mehling WE, Acree M, Stewart A, Silas J, Jones A. The Multidimensional Assessment of Interoceptive Awareness, Version 2 (MAIA-2). PLoS One. 2018;13:e0208034. doi: 10.1371/journal.pone.0208034

[2] van de Glind G, van den Brink W, Koeter MWJ, Carpentier P-J, van Emmerik-van Oortmerssen K, Kaye S, et al. Validity of the Adult ADHD Self-Report Scale (ASRS) as a screener for adult ADHD in treatment seeking substance use disorder patients. Drug Alcohol Depend. 2013;132:587–96. doi: 10.1016/j.drugalcdep.2013.04.010

[3] Abramowitz JS, Deacon BJ, Olatunji BO, Wheaton MG, Berman NC, Losardo D, et al. Assessment of obsessive-compulsive symptom dimensions: Development and evaluation of the Dimensional Obsessive-Compulsive Scale. Psychol Assess. 2010;22:180–98. doi: 10.1037/a0018260

[4] Spitzer RL, Kroenke K, Williams JBW, Löwe B. A brief measure for assessing generalized anxiety disorder: The GAD-7. Arch Intern Med. 2006;166:1092–7. doi: 10.1001/archinte.166.10.1092

[5] Rutter LA, Brown TA. Psychometric Properties of the Generalized Anxiety Disorder Scale-7 (GAD-7) in Outpatients with Anxiety and Mood Disorders. J Psychopathol Behav Assess. 2017;39:140–6. doi: 10.1007/s10862-016-9571-9

[6] Kroenke K, Spitzer RL, Williams JBW. The PHQ-9: Validity of a brief depression severity measure. J Gen Intern Med. 2001;16:606–13. doi: 10.1046/j.1525-1497.2001.016009606.x

[7] Manea L, Gilbody S, McMillan D. Optimal cut-off score for diagnosing depression with the Patient Health Questionnaire (PHQ-9): A meta-analysis. CMAJ. 2012;184:E191. doi: 10.1503/cmaj.110829

[8] Beard C, Hsu KJ, Rifkin LS, Busch AB, Björgvinsson T. Validation of the PHQ-9 in a psychiatric sample. J Affect Disord. 2016. doi: 10.1016/j.jad.2015.12.075

[9] Leckman JF, Riddle MA, Hardin M, Ort S, Swartz K, Stevenson J, et al. The Yale Global Tic Severity Scale: Initial Testing of a Clinician-Rated Scale of Tic Severity. J Am Acad Child Adolesc Psychiatry. 1989;28:566–73. doi: 10.1097/00004583-198907000-00015

[10] Haas M, Jakubovski E, Fremer C, Dietrich A, Hoekstra PJ, Jäger B, et al. Yale Global Tic Severity Scale (YGTSS): Psychometric Quality of the Gold Standard for Tic Assessment Based on the Large-Scale EMTICS Study. Front Psychiatry. 2021;12:98. doi: 10.3389/fpsyt.2021.626459

[11] Woods DW, Piacentini J, Himle MB, Chang S. Premonitory Urge for Tics Scale (PUTS): Initial psychometric results and examination of the premonitory urge phenomenon in youths with tic disorders. J Dev Behav Pediatr. 2005;26:397–403. doi: 10.1097/00004703-200512000-00001

[12] Woods D, Piacentini J, Himle M, Chang S. Premonitory Urge for Tics Scale (PUTS). J Dev Behav Pediatr. 2005;26:397–403. doi: 10.1097/00004703-200512000-00001

[13] Cavanna AE, Schrag A, Morley D, Orth M, Robertson MM, Joyce E, et al. The Gilles de la Tourette Syndrome-Quality of Life Scale (GTS-QOL): Development and validation. Neurology. 2008. doi: 10.1212/01.wnl.0000327890.02893.61

**Table 2.** Internal Reliability Consistency Estimates for MAIA-2 Subscales

| MAIA-2 Subscale | McDonald’s *ω* |
| --- | --- |
| Noticing | 0.87 |
| Not-Distracting | 0.93 |
| Not-Worrying | 0.74 |
| Attention Regulation | 0.92 |
| Emotional Awareness | 0.88 |
| Self-Regulation | 0.87 |
| Body Listening | 0.89 |
| Trusting | 0.89 |

**Table 3.** Comparison of CTD Participants with No Reported ADHD or OCD to Sex- and Age-Matched Controls – Demographics, Clinical Characteristics, and MAIA-2 Subscale Scores

| Variable | Control  (n=15) | CTD  (n=15) | Wllcoxon-Rank Sum Test for Continuous Variables |
| --- | --- | --- | --- |
| Sex (M : F) | 12 : 3 | 12 : 3 |  |
| Age (years) | 45 (30-63)^†^ | 40 (29-66) | z = 0.06 |
| Ethnicity  Hispanic or Latino  Not Hispanic or Latino | 0  15 | 0  15 |  |
| Race  Asian  Black or African American  White  Unknown / Not reported | 1  1  12  1 | 0  0  14  1 |  |
| Co-occurring conditions, self-reported  ADHD  OCD  Anxiety  Depression  Autism spectrum disorder | 0  0  1  0  0 | 0  0  6  7  0 |  |
| Adult ADHD Self-Report Screening Scale for DSM-5 (ASRS-5) | 9 (6-10) | 13 (10-15) | z = -3.1** |
| Dimensional Obsessive-Compulsive Scale (DOCS) | 13 (5-18) | 9 (0-23) | z = 0.3 |
| Generalized Anxiety Disorder-7 (GAD-7) | 1 (0-4) | 8 (2-12) | z = -2.4* |
| Patient Health Questionnaire-9 (PHQ-9) | 2 (0-4) | 9 (2-15) | z = -2.8** |
| YGTSS Total Tic Score | - | 19 (13-27) | - |
| Premonitory Urge to Tic Scale (PUTS) | - | 24 (18-29) | - |
| Gilles de la Tourette-Quality of Life Scale (GTS-QOL) | - | 25.9 (13.0-50.9) | - |
| MAIA-2 Subscales |  |  |  |
| Noticing | 2.5 (2.0-3.3) | 2.8 (2.0-3.3) | z = -0.4 |
| Not-Distracting | 2.2 (1.5-3.5) | 2.3 (1.5-3.5) | z = 0.0 |
| Not-Worrying | 3.6 (2.6-3.8) | 3.0 (2.2-3.4) | z = 1.7 (p = 0.09) |
| Attention Regulation | 2.9 (2.7-3.3) | 2.1 (1.6-3.0) | z = 1.5 |
| Emotional Awareness | 3.0 (1.8-3.2) | 3.0 (2.4-3.6) | z = -0.5 |
| Self-Regulation | 3.0 (1.8-4.0) | 2.0 (1.3-2.8) | z = 2.2* |
| Body Listening | 1.7 (1.0-3.3) | 1.3 (0.7-2.0) | z = 1.1 |
| Trusting | 3.7 (2.7-4.0) | 2.7 (2.0-4.7) | z = 0.6 |

^†^ Median (interquartile range)

* p < 0.05; ** p < 0.01; *** p < 0.001

1. **Supplementary Figures**

**Figure 1.** Bivariate Correlation Matrix for Healthy Control Participants
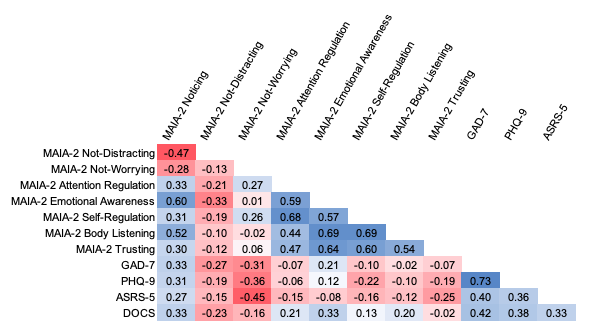


Intensity of shading reflects magnitude of Spearman rank correlation, with blue indicating positive correlation and red indicating negative correlation.

**Figure 2.** Dendrogram from Hierarchical Cluster Analysis of MAIA-2 Subscales within CTD Participants

**Figure 3.** Histograms of Regression Model Residuals

1. MAIA-2 Noticing Subscale – Full Regression Model
2. MAIA-2 Noticing Subscale – Reduced Regression Model
3. MAIA-2 Not-Worrying Subscale – Full Regression Model

1. MAIA-2 Not-Worrying Subscale – Reduced Regression Model

1. PUTS Regression Model^†^

^†^ For CTD participants only
